# Diversity Assessment of Toxic Cyanobacterial Blooms during Oxidation

**DOI:** 10.3390/toxins12110728

**Published:** 2020-11-20

**Authors:** Saber Moradinejad, Hana Trigui, Juan Francisco Guerra Maldonado, Jesse Shapiro, Yves Terrat, Arash Zamyadi, Sarah Dorner, Michèle Prévost

**Affiliations:** 1 Department of Civil, Geological, and Mining Engineering, Polytechnique Montréal, Montréal, QC H3T 1J4, Canada; Hana.trigui@polymtl.ca (H.T.); Juan-francisco.guerra-maldonado@polymtl.ca (J.F.G.M.); Sarah.dorner@polymtl.ca (S.D.); Michele.prevost@polymtl.ca (M.P.); 2 Department of Biological Science, Université de Montréal, Montréal, QC H2V 0B3, Canada; Jesse.shapiro@umontreal.ca (J.S.); Yves.terrat@umontreal.ca (Y.T.); 3 Water Research Australia (WaterRA), Adelaide, SA 5001, Australia; Arash.zamyadi@waterra.com.au; 4 BGA Innovation Hub and Water Research Centre, School of Civil and Environmental Engineering, University of New South Wales (UNSW), Sydney, NSW 2052, Australia

**Keywords:** cyanobacteria, diversity, oxidation, high throughput sequencing, *Dolichospermum*, *Microcystis*

## Abstract

Fresh-water sources of drinking water are experiencing toxic cyanobacterial blooms more frequently. Chemical oxidation is a common approach to treat cyanobacteria and their toxins. This study systematically investigates the bacterial/cyanobacterial community following chemical oxidation (Cl_2_, KMnO_4_, O_3_, H_2_O_2_) using high throughput sequencing. Raw water results from high throughput sequencing show that *Proteobacteria*, *Actinobacteria*, *Cyanobacteria* and *Bacteroidetes* were the most abundant phyla. *Dolichospermum, Synechococcus, Microcystis* and *Nostoc* were the most dominant genera. In terms of species, *Dolichospermum sp.90* and *Microcystis aeruginosa* were the most abundant species at the beginning and end of the sampling, respectively. A comparison between the results of high throughput sequencing and taxonomic cell counts highlighted the robustness of high throughput sequencing to thoroughly reveal a wide diversity of bacterial and cyanobacterial communities. Principal component analysis of the oxidation samples results showed a progressive shift in the composition of bacterial/cyanobacterial communities following soft-chlorination with increasing common exposure units (CTs) (0–3.8 mg·min/L). Close cyanobacterial community composition (*Dolichospermum* dominant genus) was observed following low chlorine and mid-KMnO_4_ (287.7 mg·min/L) exposure. Our results showed that some toxin producing species may persist after oxidation whether they were dominant species or not. Relative persistence of *Dolichospermum sp.90* was observed following soft-chlorination (0.2–0.6 mg/L) and permanganate (5 mg/L) oxidation with increasing oxidant exposure. Pre-oxidation using H_2_O_2_ (10 mg/L and one day contact time) caused a clear decrease in the relative abundance of all the taxa and some species including the toxin producing taxa. These observations suggest selectivity of H_2_O_2_ to provide an efficient barrier against toxin producing cyanobacteria entering a water treatment plant.

## 1. Introduction

The occurrence of cyanobacterial blooms in fresh-water bodies has been enhanced due to eutrophication and temperature increases [1,2]. Cyanobacterial blooms may produce and release taste and odour compounds as well as cyanotoxins into water bodies. More than 40 species of cyanobacteria are known as potentially toxic species [3,4]. Microcystin (MC), anatoxin (ATX-a), saxitoxin (STX), cylindrospermopsin (CYN) and β-Methylamino-L-alanine (BMAA) are the five major groups of cyanotoxins due to their toxicity and frequent occurrence around the world [5].

Conventional drinking water treatment plants are often challenged by the removal of cyanobacteria and cyanotoxins [6,7,8,9] including both low risk (low cell numbers in the source) and high-risk water treatment plants (high cell numbers in the source). Several studies have evaluated the removal of the cyanobacteria and their harmful metabolites using different oxidants such as chlorine, ozone and, potassium permanganate [10,11,12,13,14,15]. Pre-oxidation dampens the cyanobacterial shock before entering the drinking water treatment plant and can limit the accumulation of cyanobacteria within the plant. Chlorine and ozone are also used as primary disinfectants providing an additional oxidation barrier after the filtration to remove cyanobacterial harmful metabolites (cyanotoxins) [16,17].

Dynamic and complex behaviour of cyanobacterial blooms, including cyanotoxin production and release under various environmental conditions represents a treatment challenge. The oxidation efficiency of cyanobacteria blooms may vary according to water quality parameters (such as pH, Dissolved Organic Carbon (DOC) and the presence of other bacterial communities), cyanobacterial community shape, potential agglomeration, and the growth phase [14,18,19,20]. Cyanobacteria treatment efficiency improvement requires an understanding of the cyanobacterial composition structure in response to treatment processes [21]. Molecular methods have been used to study the fate of the microbial community, including cyanobacteria, within different conditions [5,22]. Molecular methods can overcome the challenges of microscopic cell counts such as time and qualified person requirements, as well as changes in biovolumes during analyses [23,24,25].

Molecular methods such as high throughput sequencing have been deployed to study cyanobacterial communities and identify cyanotoxin biosynthesis genes [5,26,27,28,29,30,31,32,33,34]. Diversity of the cyanobacterial community and toxigenic cyanobacteria was assessed based on the Operational Taxonomy Units (OTUs) derived from 16S rRNA gene amplification (metabarcoding) [5]. Limited studies have applied high throughput sequencing to monitor the fate of cyanobacteria during the treatment processes. Xu, Pei [35] studied the microbial community of the sludge in six different drinking water treatment plants using high throughput sequencing. Results showed that cyanobacteria were the most dominant phylum in two treatment plants with a higher level of nutrients in raw water. *Planktothrix*, *Microcystis* and *Cyindrospermopsis* were the most abundant genera and were positively correlated with the nutrient levels in raw water. Pei, Xu [36] used 16S rRNA sequencing to study the shifts in the microbial community in clarifier sludge following coagulation by FeCl_3_, AlCl_3_ and PAFC (Polyaluminium Ferric Chloride). Results revealed selective removal of the different bacterial species, as the relative abundance of the *Microcystis*, *Rhodobacter*, *Phenylobacterium* and *Hydrogenophaga* decreased in AlCl_3_ sludge compare to the FeCl_3_ and PAFC. Lower *Microcystis* abundance could be related to high Al toxicity or large and high-density floc in FeCl_3_ and PAFC, which plays a protective role for microorganisms [36]. Lusty and Gobler (2020) used 16S rRNA to evaluate the mitigation of cyanobacterial blooms using H_2_O_2_. Results showed relative persistence of *Cyanobium* and *Cylindrospermopsis* to a moderate H_2_O_2_ dose (4 mg/L); *Plankthotrix* and *Microcystis* (abundant genus) were the most sensitive genera, respectively [37].

High throughput sequencing has been widely applied to study bacterial/cyanobacterial communities. Fewer studies focused on the diversity of bacterial/cyanobacterial communities during water treatment processes. However, no study has focused on the cyanobacterial community following chemical oxidation using high throughput sequencing. Understanding the shifts and the potential selective persistence in cyanobacterial communities following oxidation is important for choosing an efficient oxidant. Thus, the objective of this study was to assess the structural composition of the cyanobacteria community following oxidation (with Cl_2_, O_3_, KMnO_4_, H_2_O_2_) using high throughput metagenomic shotgun sequencing over the seasonal bloom period.

## 2. Results and Discussion

### 2.1. Cyanobacterial Bloom Characteristics Throughout Sampling

The cyanobacteria bloom samples were collected on 5 days of the bloom period (1, 13, 15, 21 and 29 August 2018) from Missisquoi Bay (Lake Champlain) close to the water intake of the drinking water treatment plant. Cyanobacterial bloom characteristics are presented in the Table 1. DOC (Dissolved Organic Carbon) and pH did not demonstrate considerable variation throughout the sampling period. Total cell counts and biovolumes follow the same decreasing and increasing trend during the sampling period. For both parameters, the highest values were found at the beginning of the sampling period (1 August), followed by a significant drop on 13 August, where cyanobacterial cell counts decreased from 3.3 × 10^5^ to 7.8 × 10^4^ cells/mL and the biovolumes from 30.6 mm^3^/L to 4.6 mm^3^/L. The second drop was observed between 15 August and 21 August, from 1.4 × 10^5^ to 6.8 × 10^4^ cells/mL for cell count and from 9.4 to 0.3 mm^3^/L for the biovolume. According to algal cell abundance descriptors (biovolume and cell counts) during the bloom period, the main peaks of cyanobacterial bloom occurred on 1 August and to a lower extent on 15 August. The observed cell count exceeds the alert level of the 6.5 × 10^4^ cells/mL for drinking water treatment plants [38], except for the 29 August with 5.4 × 10^4^ cells/mL.

### 2.2. Variation of the Cyanobacterial Bloom Composition

**Figure 1:**
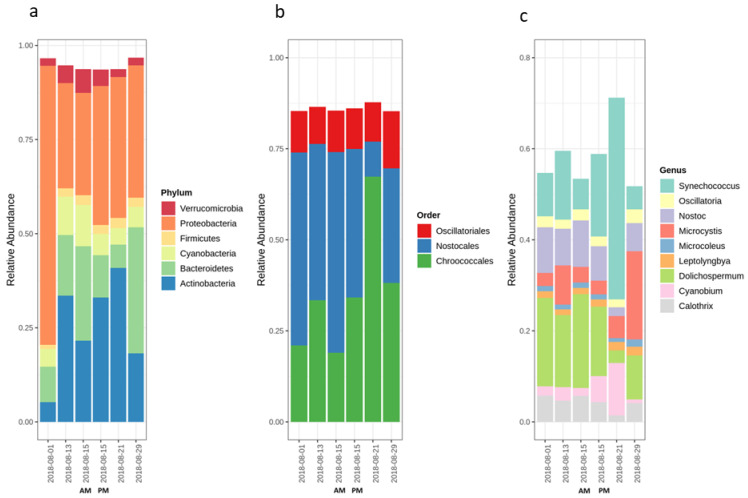
Identity of major detected bloom-associated cyanobacterial community members during the sampling period: (**a**) relative abundance of the different phylum, (**b**) the relative abundance of orders belonging to cyanobacterial phylum, (**c**) relative abundance of genera belonging to the *Nostocales*, *Chrooccocales* and *Oscillatoriales* orders.

The diversity and community variation of bacterial and cyanobacterial communities during the bloom sampling period were studied using comparative metagenomics reads levels of phylum, order and genus. The number of reads for taxonomic data was normalized by relative abundance (Figure 1).

Analysis of relative abundance of the bacterial community at the phylum level, based on high throughput sequencing data, showed that Proteobacteria, Actinobacteria, Cyanobacteria, Bacteroidetes, Firmicutes and Verrucomicrobia were the six most abundant phyla throughout the sampling period (Figure 1a). As expected, Proteobacteria was by far the most abundant phylum at the beginning of the sampling period (1 August) as has been observed by Pei et al. (2017) [36]. The high relative abundance of Proteobacteria, especially in the beginning of sampling and Bacteroidetes at the end of the sampling period, may indicate contamination of the sampling point with human/animal-associated fecal markers [39,40,41]. For the rest of the sampling dates (13, 15 and 21 August), Proteobacteria remained the predominant phylum, but at a lower extent than what was observed in the first and last days of sampling. The cyanobacteria phylum accounts for 5 to 10% of total relative abundance assigned to the phylum level in all samples. The cyanobacteria relative abundance started at 5% of the phyla, followed by an increase in the middle of the sampling (13 August and 15 August) to 10%. By the end of August, the cyanobacterial contribution in the whole bacterial community decreased to 5%.

At the order level, the cyanobacterial community was dominated by members of the *Chroococcales*, *Nostocales* and *Oscillatoriales* during the cyanobacterial bloom period (from 1 August and 29 August) (Figure 1b). The relative abundance of the *Nostocales* and *Chroococcales* varied between the different dates and even within the same day (15 August). On the other hand, *Oscillatoriales* relative abundance remained steady (Figure 1b).

Analysis at the genus level showed that within the *Chroococcales* order, the predominant genera were *Microcystis* and *Synechococcus. Synechococcus* was the dominant genus within *Chroococcales* until 21 August of the sampling period, followed by *Microcystis,* which became the dominant genus on 29 August (Figure 1c). The predominant genera in the *Oscillatoriales and Nostocales* orders were *Oscillatoria* and *Dolichospermum* (formerly known as *Anabaena*), respectively (Figure 1c). Depending on the sampling date, *Synechococcus*, *Microcystis* and *Dolichospermum* were the predominant genera during the bloom period. Our observations are consistent with short/long term investigations of cyanobacterial bloom dynamics using taxonomic cell count and metagenomics [14,21,42,43,44]. *Microcystis* and *Dolichospermum* share a spatio-temporal niche during blooms and often are dominant within the cyanobacterial community, but have distinct environmental preferences; for example, they have different responses to nutrients [45,46,47]. During this study, the abundant genus shifted from *Nostocales* members, dominated by *Dolichospermum* for the first three weeks, to the *Microcystis* genus by the end of the sampling period (29 August). The shift to *Microcystis* dominance can probably be attributed to species-specific associations between *Microcystis spp.* and associated bacteria referred to as the epibiont phenomenon. Some species within *Proteobacteria*, *Bacteroidetes* and other phyla can shelter in the *Microcystis* mucilage to avoid being grazed [48]. These species play essential roles in enhancing the environmental adaptation of *Microcystis* within the cyanobacterial bloom, like maintaining redox balance and coping with oxidative stress [48]. Our results are in accordance with the previously reported results that *Proteobacteria*, *Bacteriodetes* tend to dominate in the *Microcystis* mucilage (blooms and culture-dependent studies) [49,50]. Moreover, allelopathy may influence the successional dominance of *Microcystis* and *Dolichospermum* within aquatic systems, whereby organisms produce bioactive compounds (allelochemicals) in the environment to positively or negatively influence the growth of neighboring species [51]. The effects of these allelochemicals on cyanobacterial community distribution within the environment are connected with nutrient availability and environmental conditions [52,53,54]. Competition experiments between toxic *Microcystis* and *Dolichospermum* strains, based on the lab coculture-dependent method, showed that *Microcystis* significantly inhibited the growth of *Dolichospermum,* whereas the effects of *Dolichospermum* on *Microcystis* were minimal [53,55]. *Dolichospermum* biovolume and biomass were sharply reduced after exposure to *Microcystis* strains [56]. Further investigation is required to explain the interaction and succession of *Microcystis* and *Dolichospermum* in cyanobacterial blooms.

An interesting insight was gained considering within-day changes in bacterial community composition (morning—AM and afternoon—PM) for 15 August (Figure 1). Based on the relative abundance analysis, a shift in bacterial composition at the phylum level was observed between the two samples on 15 August. In the afternoon, *Proteobacteria* and *Actinobacteria* increased, while *Bacteroidetes* and *Cyanobacteria* decreased as compared to their initial composition at the start of the sampling in the morning. At the order level, the most abundant order shifted from *Nostocales* in the morning to *Chroococcolas* in the afternoon. At the genus level, the changes were more evident for *Synechococcus* (increase) and *Dolichospermum* (decrease). These within-day changes in bacterial composition reflect the variation in stratification and mixing patterns of the cyanobacterial community in Missisquoi bay, as shown by Ndong et al. (2014) Ndong, Bird [57]. These findings highlight the importance of timing the sample collection by considering the stratification variation in the diel cycle (morning, noon, afternoon).

### 2.3. Impact of Oxidation on Cyanobacterial Diversity

The impact of oxidation on cyanobacterial diversity was assessed by using the impact of increased CT (the terminology used in water treatment is CT, which represents the product of the oxidant concentration (C) and contact time (T) to inactivate microorganisms) on two different samples, first on 1 August, dominated by *Dolichospermum* and the second on 29 August dominated by the *Microcystis* genus. The oxidants considered Cl_2_, KMnO_4_, O_3_, H_2_O_2_ differ widely in their mode of action and their persistence [58]. As a result, CT values may vary over three orders of magnitude from 0.1 mg·min/L Cl_2_ and a maximum of 7035 mg·min/L for H_2_O_2_.

Using non-normalized data is a source of bias in statistical analyses of the effects of oxidants on the bacterial/cyanobacterial communities. Thus, the data were normalized prior to analysis. Using the common exposure unit (CT) is not representative to compare the effects of different oxidants simultaneously. A normalized oxidant exposure (relative CT) is used to compare different oxidants among each other (Relative CT for each oxidant; max CT = 1 and min CT = 0, the exposure points in between were calculated accordingly); for example, max CT obtained for H_2_O_2_, on 1 August, is 7035 mg·min/L and is considered as relative CT = 1, the control condition is considered as relative CT = 0.

The doses (concentration) and contact time (T) were selected using the product CT, which is the foundation of disinfection and oxidation in drinking water. The choice of CT also considered prior evidence showing differences in species’ resistance to the oxidant exposure (CT) [6,10,12,14,15,16,18,42,59,60]. However, all previously reported observations were based on taxonomic cell counts and not on high throughput sequencing. The applied dosages reflect drinking water industry practices using regulated treatment processes.

#### 2.3.1. Cyanobacterial Composition

**Figure 2:**
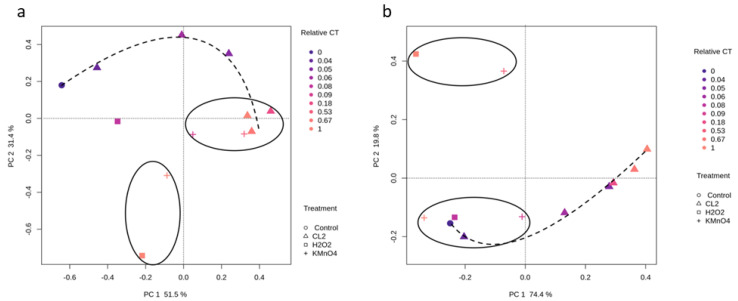
Principal components analysis (PCA) of the normalized relative abundance of comparative metagenomics reads in 1 August sample. Data are plotted following the genus-level classification (**a**) PCA analysis of bacterial community following oxidation using different common exposure units (CT), (**b**) PCA of the cyanobacterial community following oxidation using different CT.

To assess the cyanobacterial bloom composition following oxidation using KMnO_4_, Cl_2_ and H_2_O_2_, samples were taken on 1 August (Figure 2) and 29 August (Figure S1). *Dolichospermum* and *Microcystis* were the dominant genera on 1 August and 29 August, respectively.

Figure 2 shows the dissimilarity among groups of bacterial communities following oxidation at different exposures (relative CT) using Principal Component Analysis (PCA) on 1 August. Samples that appear more closely together within a PCA are assumed to be more similar in bacterial and cyanobacterial composition (Figure 2). For the bacterial community, principal axis one and principal axis two for PCA represent 51.5 and 31.4% of the variation among the samples, respectively. High-relative CT exerted clustering of two groups, the first one includes samples after Cl_2_ and KMnO_4_ oxidation and the second one encompasses KMnO_4_ and H_2_O_2_ oxidation. Chlorinated samples showed a clear progressive shift (as the relative CT increased) in the bacterial community.

On the other hand, the KMnO_4_ and H_2_O_2_ induce large shifts as relative CT increases. Bacterial and cyanobacterial composition similarity following the different oxidation on 29 August (*Microcystis*) is presented in Figure S1. Like the 1 August result, a large shift in the bacterial community composition following H_2_O_2_ oxidation was observed for 29 August. On the other hand, KMnO_4_ results showed similar bacterial composition on 29 August.

For the cyanobacterial community (1 August), principal axis one, and principal axis two for PCA represent 74.4 and 19.8% of the variation among the samples, respectively (Figure 2b). A clear trend in the cyanobacterial composition variation is observed following chlorination. Cyanobacterial composition after exposure to Cl_2_ (low relative CT < 0.05), KMnO_4_ (low and high relative CT), H_2_O_2_ (low relative CT = 0.08) oxidation clustered in the same group with the control condition. High-relative (CT = 1) using H_2_O_2_ and (mid-relative = 0.53) KMnO_4_ are grouped in a distinct second cluster, revealing similar cyanobacterial assemblages. This is in contrast with cyanobacterial/bacterial community trends following chlorination that display a progressive shift. Differences between the observed trends for the three oxidants were expected because of different mechanisms of actions and kinetics, persistence and selectivity. Cell count based studies show progressive shifts following oxidation [12,14]. Moreover, selective cyanobacteria oxidation has been demonstrated in the lab [11,60], in the field [37,61], and in drinking water treatment plants [8,62].

**Figure 3:**
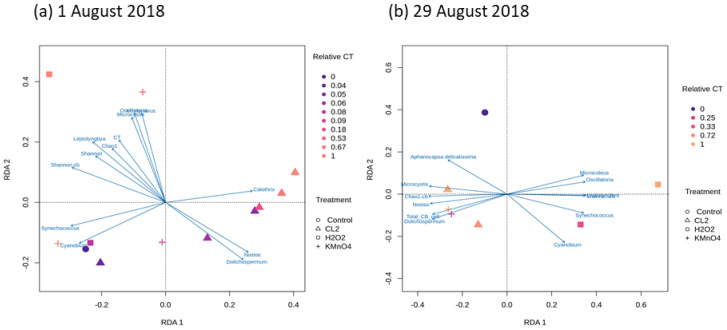
Redundancy analysis (RDA) of oxidant effect on cyanobacterial diversity and the cyanobacterial community at genus level Cl_2_ (0.6 mg/L), KMnO_4_ (5 mg/L), H_2_O_2_ (10 mg/L) (**a**) 1 August 2018 (**b**) 29 August 2018.

Our results enlighten the different oxidation impacts on the bacterial community between the Cl_2_ and H_2_O_2_ oxidation. For 29 August, which was dominated by the *Microcystis* genus, KMnO_4_ results revealed similar cyanobacterial composition. However, a large variation in cyanobacterial composition following H_2_O_2_ was observed (e.g., the samples from the 1 August, Figure 3).

Redundancy analysis (RDA) was used to explore the correlation between the relative abundance of nine dominant cyanobacterial genera (60% of the cyanobacterial community) observed in 1 August or 29 August samples and the different oxidant exposures (Figure 3). The relationship between the CT and diversity indexes (Shannon and Chao1) varies between the two dates. On 1 August, both diversity indices increase with CT, while on 29 August only the Richness index (Chao1) increased with CT. In general, the diversity of the cyanobacterial community increased due to oxidation of the dominated genus, as discussed further in detail for each oxidant.

For 1 August (*Dolichospermum* dominant), RDA analysis establishes an inverse correlation between the *Dolichospermum* genus and a wide range of chlorine exposures with relative CT > 0.04. Chlorination appears to have a lesser impact on the *Dolichospermum* genus than the *Microcystis*, possibly because of its abundance. On the other hand, no such relationship is observed between *Dolichospermum* and KMnO_4_ exposure (relative CT = 1) and high H_2_O_2_ exposure (relative CT = 1), suggesting that these oxidants at these exposure levels had a negative impact on the *Dolichospermum* genus persistence within the community. Furthermore, for KMnO_4_ at relative CT > 0.53, *Microcystis*, *Synechococcus*, and *Leptolyngbya* are less impacted by oxidation. Figure 3b shows the effect of the different oxidants on the cyanobacterial community of 29 August, when *Microcystis* genus is dominant. An immediate shift from the control is observed for all oxidants and for all relative CT. Unlike 1 August, the Shannon diversity is no longer correlated with *Dolichospermum*. These differences could reflect the morphological differences between the dominant genera (*Microcystis* vs. *Dolichospermum)* as a unicellular aggregate that are less resistant to oxidation as compared to filaments [63]. Chlorine and KMnO_4_ exposures had a low impact on *Microcystis* and *Dolichospermum*. On the other hand, any H_2_O_2_ exposure causes a reduction in *Dolichospermum* and *Microcystis,* more so on 29 August. Removal of *Microcystis* using H_2_O_2_ was shown by Lusty and Gobler. (2020) and is in accordance with our observations of diverging correlation between Chao1 and *Microcystis* [37].

Figure S2 shows the cyanobacterial composition following oxidation at genus level. Results show no significant variation in the relative abundance of *Dolichospermum* and *Microcystis* following Cl_2_, KMnO_4_ oxidation. Thus, no relative persistence to oxidation was observed at the genus level. On the other hand, high H_2_O_2_ exposure caused a decline in both *Dolichospermum* and *Microcystis* genus, which demonstrates the effect of high H_2_O_2_ exposure on cyanobacterial removal.

#### 2.3.2. Effect of Oxidation on Cyanobacterial Community Richness and Diversity

**Figure 4:**
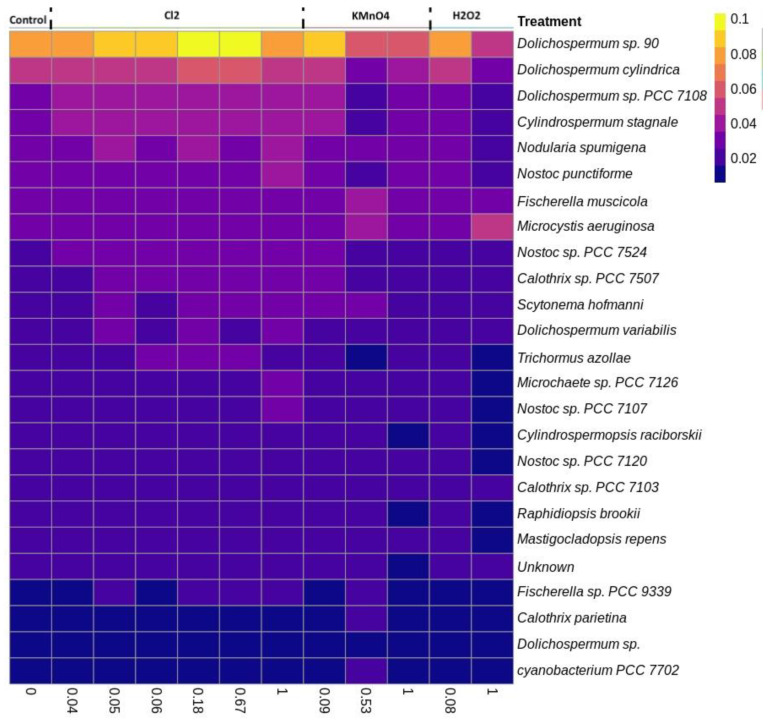
Cyanobacterial species heat map following the oxidation using Cl_2_ (0.6 mg/L), KMnO_4_ (5 mg/L), H_2_O_2_ (10 mg/L) (1 August 2018).

The diversity of the cyanobacterial composition following oxidation (1 August) at the species level is presented in Figure 4 (top 25 most abundant species). *Dolichospermum sp.90* was the dominant species in control conditions, and its relative abundance increases following chlorination and decreases after KMnO_4_ and H_2_O_2_. Although no trends in relative abundance were seen at the genus level (Figure S2), a similar trend can be seen for the three species present *Dolichospermum sp.90, Dolichospermum cylindrica* and, *Dolichospermum sp. PCC7108*. Regardless of the oxidant *Dolichospermum sp.90* remains the dominant species. Following the KMnO_4_ and H_2_O_2_ oxidation, *Dolichospermum sp.90* was still the dominant species; its relative abundance declined compared to the control. The relative abundance of *Microcystis aeruginosa* is not impacted by chlorine but increases with KMnO_4_ and H_2_O_2_ exposure, confirming selective removal of *Microcystis* showed by Lusty and Gobler. (2020) [37].

In the 29 August samples, *Microcystis aeruginosa* was the most abundant species (Figure S3); its relative abundance increased after chlorination and KMnO_4_ oxidation (as the chlorine exposure increased) and decreased after H_2_O_2_ oxidation as compared to the control condition. Despite the very low relative abundance of *Dolichospermum sp.90, Dolichospermum cylindrica* and *Dolichospermum sp. PCC7108*, similar trends were observed on 1 August (when *Dolichospermum* was abundant).

**Figure 5:**
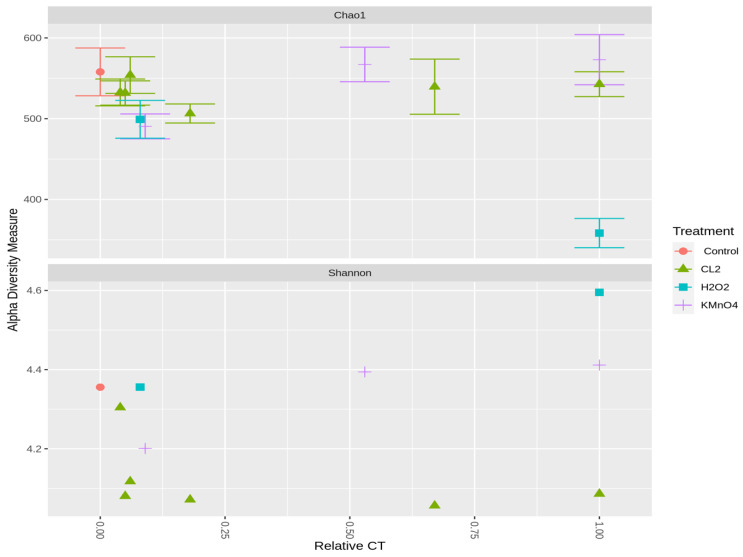
Alpha diversity measures of the cyanobacterial community following oxidation Cl_2_ (0.6 mg/L), KMnO_4_ (5 mg/L), H_2_O_2_ (10 mg/L) (1 August 2018).

The community richness and diversity indices for each treatment for 1 August and 29 August samples are illustrated in Figure 5 and Figure S4. Shannon and Chao1 show a small decline following chlorination in comparison with the control, while they increase slightly following KMnO_4_ exposure. The total cell numbers following KMnO_4_ decreased by up to 63% for high KMnO_4_ exposure (Figure S7). A remarkable decrease in richness is observed at high relative CT of H_2_O_2_, while the diversity (Shannon index) increases. The decline in the richness index could be the result of some less abundant species no longer identified. Indeed, total cell counts following H_2_O_2_ relative CT = 1, decreased by more than 50% (Figure S6). The same trends in the alpha diversity measurements are observed in the last week of the sampling (29 August), where the *Microcysits* were the most abundant genus (Figure S4).

### 2.4. Cyanobacterial Community Assessment Following Oxidation; Longitudinal Study

The induced changes of cyanobacterial composition structure following the oxidation (Cl_2_, KMnO_4_, O_3_ and, H_2_O_2_) are assessed separately. The analysis was performed at the genus and species level.

#### 2.4.1. Chlorination (Cl_2_)

**Figure 6:**
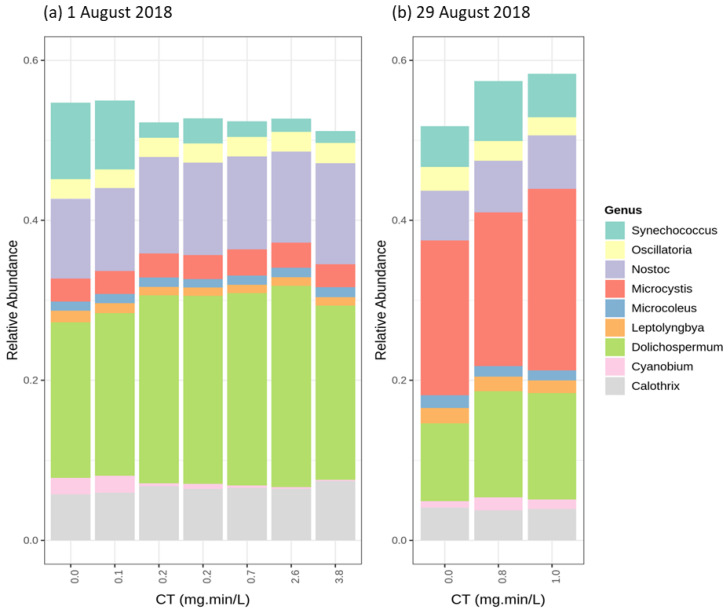
The relative abundance of the most abundant genus following chlorination (0.6 mg/L and 0.2 mg/L) (**a**) 1 August 2018 (*Dolichospermum* genus abundant) (**b**) 29 August 2018 (*Microcystis* genus abundant).

The chlorination experiments were conducted on 1 August (Figure 6a), and 29 August (Figure 6b). In the first chlorination trial (1 August), the abundant genera were *Dolichospermum* and *Nostoc*, representing approximatively 20% and 10% of the cyanobacterial community, respectively (Figure 6a). Figure 6 shows a limited effect of chlorination on the relative abundance of all cyanobacteria genera except *Synechococcus.* In addition, taxonomic cell counts show decrease (up to 30%) in total cyanobacteria cell counts for the trial on 1 August and limited variation for the 29 August trial (15% variation) (Figure S5). In terms of the species, *Dolichospermum sp.90* and *Dolichospermum cylindrica* were dominant for the 1 August trial. For the 29 August trial, *Microcystis aeruginosa* was the dominant species, followed by *Dolichospermum sp.90* (Figure S6). In all trials, as chlorination exposure increased, the relative abundance of the abundant species, either *Dolichospermum sp.90 or Microcystis aeruginosa*, increased slightly. Moreover, the relative abundance of *Dolichospermum sp.90* increased as *Microcystis aeruginosa* did on 29 August. Chlorination results show that *Dolichospermum* species and *Microcystis aeruginosa* are relatively more persistent than the other species.

#### 2.4.2. Potassium Permanganate (KMnO_4_)

**Figure 7:**
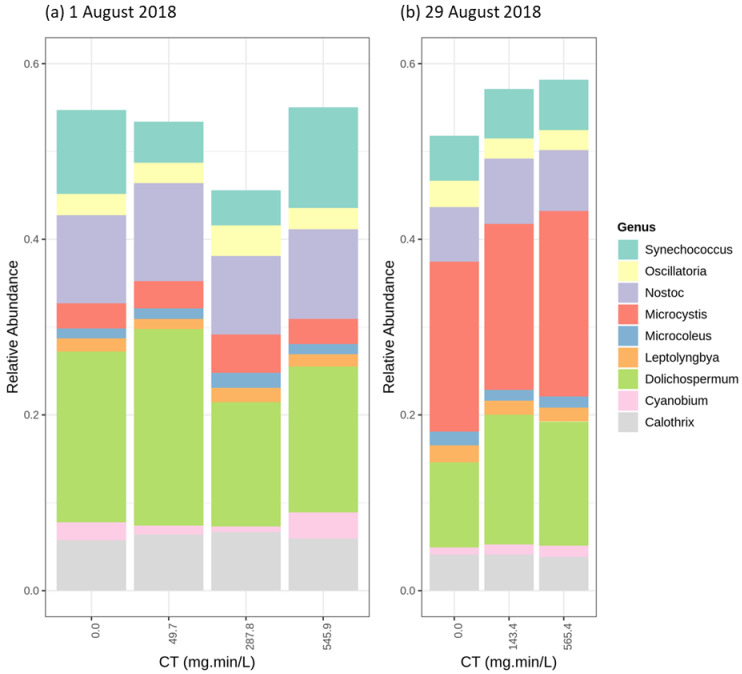
Relative abundance of the most abundant genus following KMnO_4_ (5 mg/L) oxidation (**a**) 1 August 2018 (*Dolichospermum* genus abundant) (**b**) 29 August 2018 (*Microcystis* genus abundant).

The relative abundance of the different genera following oxidation using permanganate shows limited variation for both KMnO_4_ tests (Figure 7). Total cell counts decreased (up to 57%) in the first trial (1 August) and remained stable in the second KMnO_4_ trial (less than 1% variation) (29 August). Total cell counts following the first KMnO_4_ trial showed a decrease at 278 mg·min/L (Figure S7). The relative abundance of the *Dolichospermum sp.90* increased slightly, whether it was the abundant species or not, suggesting the relative persistence of the *Dolichospermum sp.90* during KMnO_4_ oxidation (Figure S8).

#### 2.4.3. Ozonation (O_3_)

**Figure 8:**
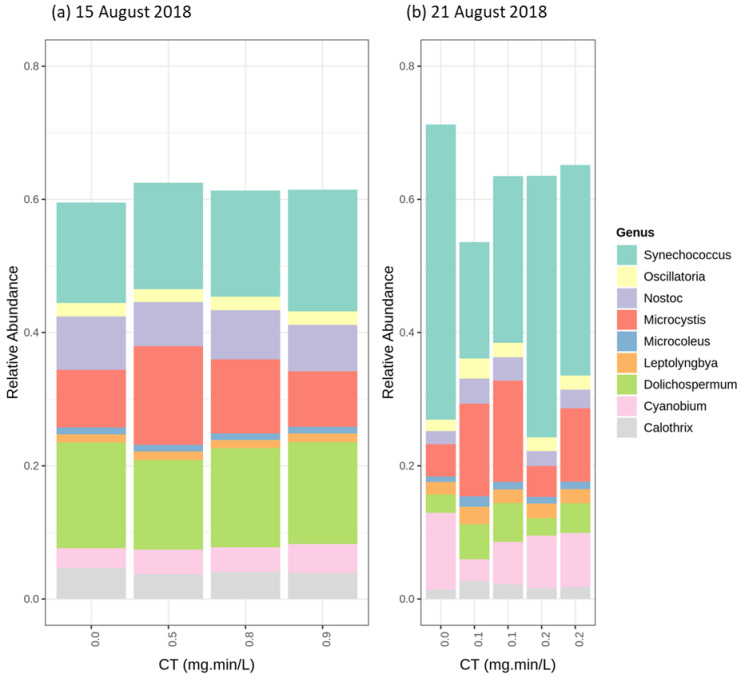
Relative abundance of the most abundant genus following O_3_ (0.3 mg/L and 0.1 mg/L) oxidation (**a**) 15 August 2018 (*Dolichospermum* genus Abundant), (**b**) 21 August 2018 (*Synechococcus* genus abundant).

The first and second ozonation trials were performed on 15 August (*Dolichospermum* most abundant genus) and 21 August (*Synechococcus* most abundant genus). Cyanobacterial community results following ozonation in the second trial (at genus level—Figure 8) showed a decline of the relative abundance of *Synechococcus,* followed by an increase for *Microcystis,* as compared to the control condition. However, no significant variation was observed for the relative abundance of the different genera in the first ozonation trials. Furthermore, total cyanobacteria cell counts revealed no significant change for both ozonation tests (up to 15%) (Figure S9). In the control condition of the 15 August ozonation trial, *Microcystis aeruginosa* was the dominant species, followed by *Dolichospermum sp.90* (Figure S10). *Dolichospermum sp.90* was not the dominant species, but it remained intact following ozonation. Although *Cyanobioum gracil* and *Synechococcus sp.* were the dominant species in the 21 August trial control, *Microcystis aeruginosa* became the dominant species following ozonation. At the low dosage applied, only secondary oxidation radical by-products are likely to react with cyanobacterial cells. Under these soft ozonation conditions, unicellular *Cyanobium gracil* and *Synechococcus sp.* cells were more susceptible than *Microcystis aeruginosa*.

#### 2.4.4. Hydrogen Peroxide (H_2_O_2_)

**Figure 9:**
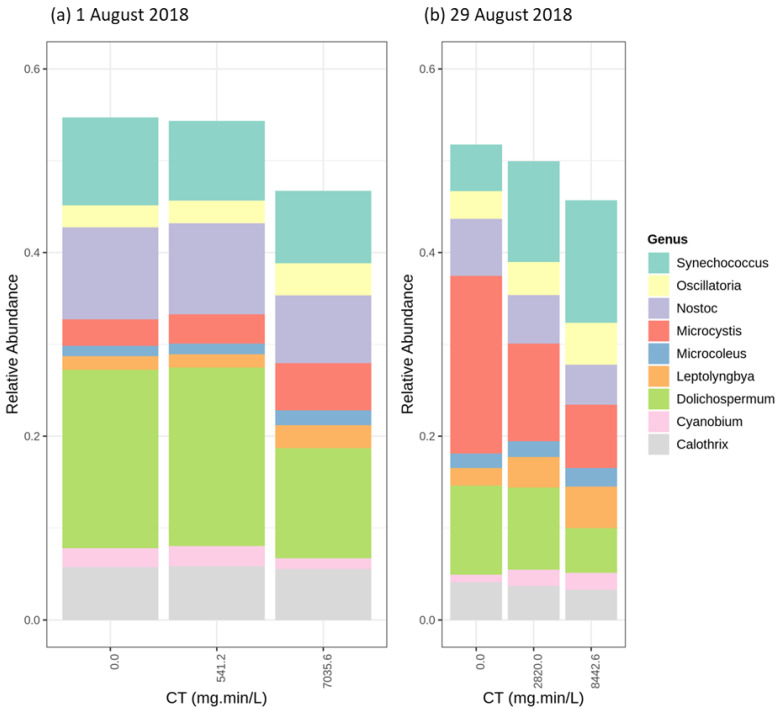
Relative abundance of the most abundant genus following H_2_O_2_ (10 mg/L) oxidation on (**a**) 1 August 2018 (*Dolichospermum* genus abundant) (**b**) 29 August 2018 (*Microcystis* genus abundant).

The *Dolichospermum*/*Dolichospermum sp.90* and *Microcystis/Microcystis aeruginosa* were the most abundant genus/species on 1 August and 29 August H_2_O_2_ oxidation, respectively. The relative abundance of the *Dolichospermum* declined following H_2_O_2_ exposure of CT = 7035 mg·min/L. *Microcystis* relative abundance decreases by more than 10% at 8442 mg·min/L exposure of H_2_O_2_ on 29 August experiment (Figure 9). At the same H_2_O_2_ exposure, *Dolichospermum* decreased by 5%. Total cyanobacteria cell counts declined following H_2_O_2_ oxidation for both dates: 52% decrease on 1 August and 49% decrease on 29 August (Figure S11). The relative abundance of the dominant species (*Dolichospermum sp.90* 1 August and *Microcystis aeruginosa* 29 August) decreases after H_2_O_2_ oxidation. The relative abundance of *Microcystis aeruginosa* in the H_2_O_2_ trial on 1 August increased after high H_2_O_2_ exposure. *Microcystis aeruginosa* was susceptible to H_2_O_2_ oxidation when abundant (as the relative abundance decreased), but it persists as an abundant species. Our results are in accordance with Lusty and Gobler. (2020) [37]. Figure S12 unveils higher susceptibility of *Dolichospermum* species to high H_2_O_2_ exposure as compared to *Microcystis aeruginosa*. Our results show the effect of high H_2_O_2_ exposure on the cyanobacteria species, which is in accordance with previous studies [61,64].

Cyanobacterial composition analysis at species level exhibits relative persistence of different *Dolichospermum* species and *Microcystis aeruginosa* when it is abundant following soft-chlorination, soft ozonation and permanganate oxidation. This result is in accordance with the previous studies, which were mainly based on the cell count and the lab-cultured species [10,12,14,58,59]. Our results highlight the ability of hydrogen peroxide to decrease the relative abundance of different taxa, including the toxin-producing taxa of interest. H_2_O_2_ selectivity provides an efficient barrier against toxic cyanobacteria entering a drinking water treatment plant.

### 2.5. Comparison of the Microscopic Cell Count vs. High Throughput Sequencing Results

The taxonomic cell count results (genus) from the chlorination on 1 August and ozonation on 15 August are presented in Figure S13. The observed genera from microscopic cell counts do not completely match with high throughput sequencing results as *Aphanocapsa* and *Aphanothece* were only reported by taxonomic cell count. Misclassification at the genus level is less common than for species level; microscopic cell counts showed that *Dolichospermum spiroids*, *Aphanocapsa delicatissma*, *Aphanotheche clathrate brevis* and *Aphanocapsa holistca* were the most abundant species following chlorination and ozonation. However, high throughput sequencing did not identify them as an abundant species. The abundant species from high throughput sequencing were *Microcystis aeruginosa, Dolichospermum Sp.90* and, *Dolichospermum cylindrica* (Figures S6 and S10). Despite the potential of the microscopic cell count to provide absolute quantitative data such as cells/mL and biovolume, it has some drawbacks.

The differences in community composition structure retrieved from the microscopic taxonomic cell counts and high throughput sequencing could be the result of the limitations inherent to these methods. For high throughput sequencing, these limitations include incomplete DNA sequencing libraries or using different libraries to identify genus and species. In the case of microscopic taxonomic cell counts, several sources of uncertainty and error have been identified [65,66]. The morphological similarity among cyanobacteria taxa may lead to overlooking or misidentifying cyanobacteria under the microscope, especially low abundant species [5,28]. Few genomes might be available for some taxa identified by taxonomic cell count (e.g., *Aphanocapsa* and *Aphanothece*), which may result in low relative abundance of these taxa using high throughput sequencing. Additionally, oxidation may further hinder the ability to identify cells because of its impact on the cell structure. Oxidation at higher dosages can cause significant morphological deformation of the cyanobacteria species, especially for H_2_O_2_ and KMnO_4_ in our study [63]. Flow cytometry conducted using the method described by Moradinejad et al. (2019) showed partial membrane damage (up to 80%) under the soft-oxidation conditions performed in this study (data not shown) [63].

This study is the first to provide insights into the impact of different pre-oxidants (doses and contact time) on the diversity and cyanobacterial community composition. The experimental design focused on CTs at which the shifts occurred, in order to provide actionable results for water utilities. Additional investigation would be beneficial to extend our observations to other water bodies. In addition, using assembly/binning method is suggested for future studies to provide a more accurate view of cyanobacterial genus/species.

## 3. Conclusions

A comparison of the microscopic vs. high throughput sequencing results demonstrates the ability and robustness of high throughput sequencing to fully reveal a wide diversity of cyanobacterial communities in response to oxidant stress.

Results from longitudinal sampling over a bloom period of 4 weeks by high throughput sequencing highlight quick composition and abundance shifts in the cyanobacterial communities that occur within a day. High throughput sequencing revealed clearer shifts during a bloom from an initial dominance of *Dolichospermum*/*Dolichospermum Sp.90* toward a late summer dominance by *Microcystis*/*Microcystis aeruginosa.*

Overall, pre-oxidation caused deeper changes in the diversity of whole bacterial communities, especially proteobacteria, than was observed for the cyanobacterial community. Such changes should be considered when assessing the impact of using oxidants for onsite source control.

Depending on the oxidants used, alpha diversity indexes (Shannon and Chao1) showed that oxidation resulted in different structural composition shifts within bacterial and cyanobacterial communities.

Soft-chlorination using dosages 0.2 and 0.6 mg/L that were typically used for pre-oxidation caused a progressive shift in the bacterial and cyanobacterial communities with increasing CTs (0–3.8 mg·min/L). The results using KMnO_4_ (5 mg/L) and H_2_O_2_ (10 mg/L) induced larger and distinct shifts in the structural composition of bacterial and cyanobacterial communities.

Regardless of the significant differences in community distribution shifts caused by different oxidants, some toxin producing species could persist after oxidation whether they were dominant species or not.

Soft-chlorination results revealed that *Dolichospermum sp.90* was relatively persistent with increasing CTs whether it was the dominant species or not. Relative persistence of *Dolichospermum sp.90* was also observed within KMnO_4_ oxidation with increasing KMnO_4_ exposure, regardless of the dominant species.

Soft-ozonation using dosage 0.1–0.3 mg/L results showed the relative persistence of *Microcystis aeruginosa* (the dominant species) with increasing ozone exposures (0–0.9 mg·min/L).

Only pre-oxidation with H_2_O_2_ (10 mg/L) caused a clear decrease in the relative abundance of all taxa and some species including the toxin-producing taxa of interest. As such, H_2_O_2_ would provide an effective first barrier against toxin producing cyanobacteria entering the drinking water treatment plant.

Selection of the most effective pre-oxidant for drinking water purposes should be made considering its impact on the cyanobacterial community structure/diversity, prevention of disinfection by-product formation and other drivers such as color and the presence of taste odor compounds.

## 4. Material and Methods

### 4.1. Sampling Site Description

The oxidation tests were conducted using natural bloom samples. Cyanobacterial bloom samples were collected and transferred to the laboratory from the Bedford water treatment plant intake (Missisquoi Bay—Lake Champlain) in southern Quebec, Canada (45°02′22.0″ N 73°04′40.5″ W). Sampling was performed during the bloom season from 1 August to 29 August 2018. Several cyanobacterial blooms have been documented for the Lake Champlain in previous years [14,21,42,43].

### 4.2. Chemicals and Reagents

A free chlorine stock of 2000 mg/L was freshly prepared from sodium hypochlorite (5.25%) on the day of the experiment. Free chlorine residual was measured using an N,N-diethyl-p-phenylenediamine (DPD) colorimetric method based on Standard Methods (SM) 4500-Cl G [67]. Samples were dosed at room temperature (22 °C). A stock of sodium thiosulfate 3000 mg/L was used to quench the chlorine samples at a dose of 1.1 mg/L per 1 mg/L chlorine.

A potassium permanganate (KMnO_4_) stock solution of 5000 mg/L was prepared by dissolving KMnO_4_ crystals into the ultrapure water. A DPD colorimetric method ratio of 0.891 KMnO_4_/Cl_2_ SM 4500-Cl G was used to determine the KMnO_4_ residual [67]. An amount of 1.2 mg/L sodium thiosulfate per 1 mg/L KMnO_4_ was used to quench further oxidation with KMnO_4_.

A bench-scale ozone generator (details in [14]) was used to prepare an ozone stock solution 50–60 mg/L. SM 4500-O_3_ was used to determine the stock and ozone residual concentration [14]. An ozone stock solution (50–60 mg/L) was prepared with gaseous ozone using a bench-scale ozone generator (additional details in [14]). Ozone stock concentration and residual ozone in water samples were measured using SM 4500-O_3_ [14]. An amount of 1.6 mg/L sodium thiosulfate per 1 mg/L ozone was used to quench the ozonation samples.

Stabilized hydrogen peroxide (30%, Sigma Aldrich, MO, USA) was used to prepare the Hydrogen peroxide (H_2_O_2_) stock (10 g/L). A colorimetric test kit (Chemetrics K-5510, Midlands, VA, USA) was performed to measure the hydrogen peroxide residual. Moreover, 1.2mg/L sodium thiosulfate per 1 mg/L H_2_O_2_ was used to quench further oxidation with H_2_O_2_.

The selected oxidant doses and contact times are presented in Table 2. The doses (concentration) and contact time have been selected based on the common pre-oxidation doses used in drinking water treatment intake.

Oxidant exposures, concentration vs. contact time, (CT) were calculated using Equation (1):

(1) $$CT=\int_{0}^{t}\left[Oxidant\right]dt=\frac{C_{0}}{k_{decay}}(e^{k_{decay}t}-1)$$

where *k_decay_* (min^−1^) is the first-order decay rate, *t* (min) is the exposure time, and *C*_0_ (mg/L) is the initial concentration of oxidant at time zero. The selection of oxidant dose was based on the commonly used pre-oxidation doses in the operation of the drinking water treatment plant.

### 4.3. DNA Extraction, Metagenomic Preparation, Sequencing and Bioinformatic Analysis

Total nucleic acid was extracted from the frozen filters (after filtration, samples were quickly transferred to −80 °C before DNA extraction) using an RNeasy PowerWater Kit (Qiagen Group, Germantwon, MD, USA) with modification. Before the extraction, 200 µL of nuclease-free water and 5 µL of TATAA Universal DNA spike II (TATAA Biocenter AB) were added to the filters to evaluate DNA extraction yields using RT-qPCR. RNeasy PowerWater Isolation kit solution PM1 was used to lyse the cells along with Dithiothreitol (DTT), which prevents disulfide bonds forming residues of proteins. A total volume of 60 µL nuclease-free water provided with the kit was used to elute the total nucleic acid, of which 30 µL of DNA (with minimum 1 ng of DNA) extracts were stored at −20 °C. DNA was subsequently purified with the Zymo Kit (Zymo Research, Irvine, CA, USA) according to the manufacturer’s instructions. Each DNA sample was resuspended in 60 µL of nuclease-free water and quantified with a Qubit v.2.0 fluorometer (Life Technologies, Burlington, ON, Canada). A volume of 30 µL DNA was sent for pyrosequencing (Roche 454 FLX instrumentation with Titanium chemistry) to the Genome Quebec.

An Illumina NovaSeq 6000 platform using S4 flow cells was applied to sequence DNA libraries. A home-made bioinformatic pipeline was used for further analysis of Paired-end raw reads of 150 base pairs (bp) as follows. First, raw reads trimming quality was performed using the SolexaQA v3.1.7.1, default parameters [68]. Further analyses were carried out on the trimmed reads shorter than 75 nt. An in-house script, based on the screening of identical leading 20 bp, was used to remove artificial duplicates. Gene fragments were predicted using FragGeneScan-Plus v3.0 based on the trimmed high-quality reads [69]. Then, predicted fragments of protein were clustered at 90% similarity level using cd-hit v4.8.1 [70]. A diamond engine was used for similarity search on the M5nr database based on a representative of each cluster (https://github.com/MG-RAST/myM5NR). Best hits (minimal e-value of 1 × 10^−5^) combined with the last common ancestor approach were used to assess the taxonomic affiliation of protein fragments. It should be mentioned that the annotation process uses a read-mapping process of small gene fragments of encoding proteins on a large database of proteins. One gene fragments encoding protein in the database could match with multiple species or strains.

### 4.4. DOC and Cyanobacteria Cell Count

Pre-rinsed 0.45 µm membrane filters (Supor 45 µm, 47 m, PES PALL, Port Washington, NY, USA) and carbon-free glass vials were used for Dissolved Organic Carbon (DOC) samples. DOC measurements were performed via a 5310 total organic carbon analyzer (Sievers Analytical Instruments, Boulder, CO, USA). An inverted microscope with 20× magnification was used to perform cell count samples preserved with Lugol’s Iodine [71,72].

### 4.5. Statistical Analysis

All analyses were performed using a custom bioinformatics pipeline implemented in R (v.3.6,2, RStudio, Inc., Boston, MA, USA), phyloseq (V.1.28.0) to visualize the community composition at phylum (all bacteria reads), order, and genus (cyanobacteria reads) [73]. The twenty-five most abundant cyanobacteria species were visualized using pheatmap (v.1.0.12) [74]. Then, the alpha diversity metrics were estimated using phyloseq’s estimate richness function (Shannon and Chao1). Taxonomic data were normalized by the centred log-ratio transformation using easy CODA (v.0.31.1) [75]. The beta-diversity was analyzed using the vegan package (v.2.5-6), where the similarity matrices were calculated based on the Euclidean distance [76]. The homogeneity of variances of normalized data related to each oxidant was analyzed before building the model. A Redundancy Analysis (RDA) constrained ordination to each oxidant applied to the cyanobacteria genus and tested by the permutation test (>95% significance).

## Figures and Tables

**Table 1:** Cyanobacterial bloom characteristics.

Sampling Date	DOC (mg/L)	pH	Cell Count (cells/mL)	Biovolume (mm^3^/L)
1 August 2018	5.9	7.6	3.3 × 10^5^	30.6
13 August 2018	5.8	7.3	7.8 × 10^4^	4.6
15 August 2018	5.5	7.4	1.4 × 10^5^	9.4
21 August 2018	4.9	7.4	6.8 × 10^4^	0.3
29 August 2018	5.6	7.5	5.4 × 10^4^	2.1

**Table 2:** Experimental plan.

Oxidant	Water Type	Oxidant Dose (mg/L)	Contact Time
Cl_2_	Real Bloom, Missisquoi Bay, Quebec, Canada	0.2 0.6	1 min 2 min 5 min 10 min 60 min 120 min
O_3_		0.1 0.3	1 min 2 min 5 min 10 min
KMnO_4_		5	30 min 60 min 120 min
H_2_O_2_		10	6 h 24 h

## Supplementary Materials

The following are available online at https://www.mdpi.com/2072-6651/12/11/728/s1. Figure S1: Principal components analysis (PCA) of the normalized relative abundance of comparative metagenomics reads in 29 August 2018 sample. Data are plotted following the genus-level classification (a) PCA analysis of bacterial community following oxidation using different CT (b) PCA of the cyanobacterial community following oxidation using different CT. Figure S2: Relative abundance of the most abundant genus following the oxidation using Cl_2_, KMnO_4_, H_2_O_2_ (1 August 2018 abundant: *Dolichospermum*). Figure S3: Cyanobacterial Species heat map following the oxidation using Cl_2_, KMnO_4_, H_2_O_2_ (29 August 2018 abundant: *Microcystis*). Figure S4: Alpha diversity measures of cyanobacterial community following oxidation Cl_2_, KMnO_4_, H_2_O_2_ (29 August 2018, abundant genus: *Microcystis*). Figure S5: Total cyanobacteria cell counts following chlorination for 1 August 2018 trial and 29 August 2018 trial. Figure S6: Cyanobacterial species heat map following the chlorination (a) 1 August 2018 trial, (b) 29 August 2018 trial. Figure S7: Total cyanobacteria cell counts following the permanganate oxidation for 1 August 2018 trial and 29 August 2018 trial. Figure S8: Cyanobacterial Species heat map following the oxidation using KMnO_4_ (a) 1 August 2018 trial, (b) 29 August 2018 trial. Figure S9: Total cyanobacteria cell counts following the O_3_ oxidation for 15 August 2018 trial and 21 August 2018 trial. Figure S10: Cyanobacterial Species heat map following the oxidation using O_3_ (a) 15 August 2018 trial, (b) 21 August 2018 trial. Figure S11: Total cyanobacteria cell counts following the H_2_O_2_ oxidation for 1 August 2018 trial and 29 August 2018 trial. Figure S12: Cyanobacterial Species heat map following the oxidation using H_2_O_2_ (a) 1 August 2018 trial (b) 29 August 2018 trial. Figure S13: Relative abundance of cyanobacteria species (via light Microscopy) following oxidation (a) O_3_ second trial (15 August 2018) (b) Cl_2_ first trial (1 August 2018).
