# Supplementary material for: Diversity Assessment of Toxic Cyanobacterial Blooms during Oxidation
Source: Toxins (Basel). 2020 Nov 20;12(11):728. doi: 10.3390/toxins12110728 (PMC7699887; doi:10.3390/toxins12110728)
Supplement: Supplementary file 1 [file toxins-12-00728-s001.pdf]

Supplementary Materials: Diversity Assessment of Toxic Cyanobacterial Blooms during Oxidation

Saber Moradinejad, Hana Trigui, Juan Francisco Guerra Maldonado, Jesse Shapiro, Yves Terrat, Arash Zamyadi, Sarah Dorner and Michèle Prévost

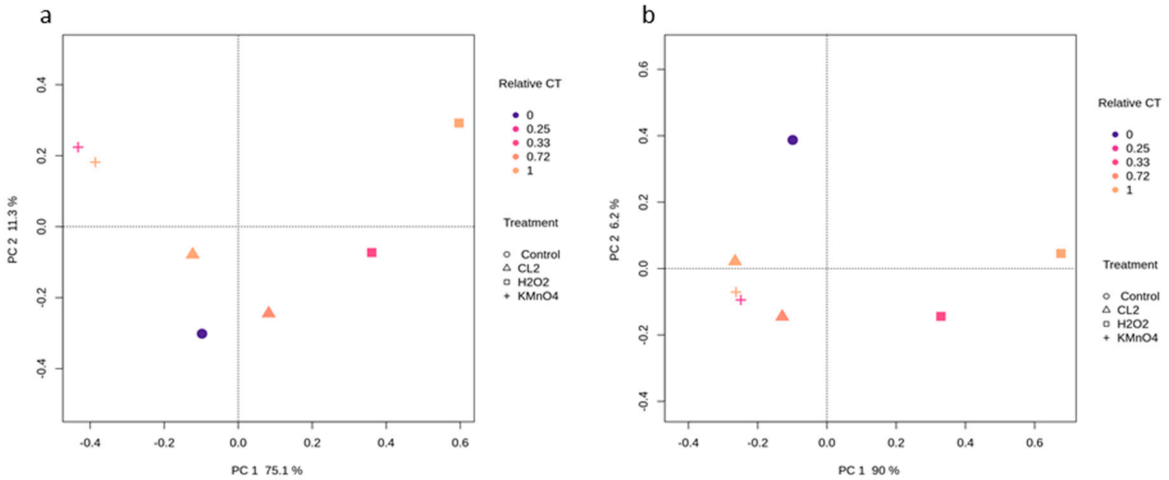

Figure S1. Principal components analysis (PCA) of the normalized relative abundance of comparative metagenomics reads in 29 August 2018 sample. Data are plotted following the genus-level classification (**a**) PCA analysis of bacterial community following oxidation using different CT (**b**) PCA of the cyanobacterial community following oxidation using different CT.

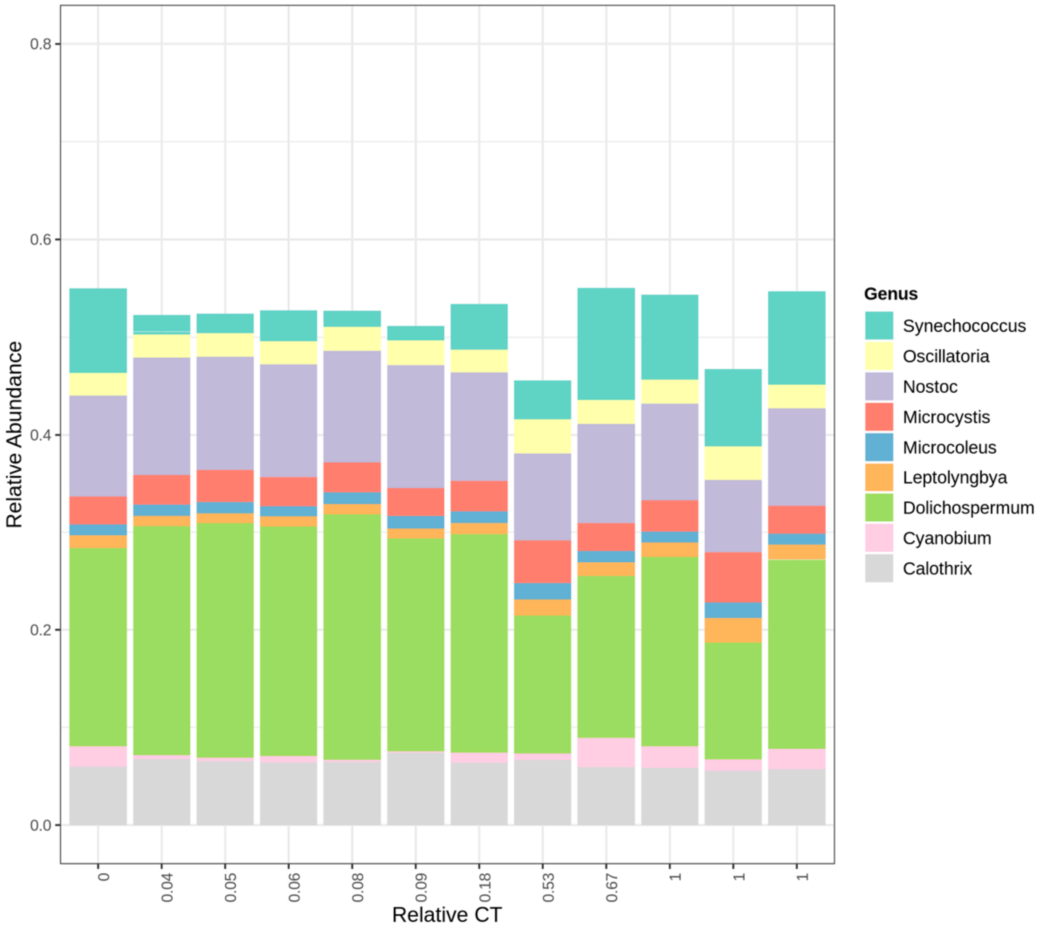

Figure S2. Relative abundance of the most abundant genus following the oxidation using Cl_2, $KMnO_4$, H_2O_2 (1 August 2018 abundant: *Dolichospermum*).

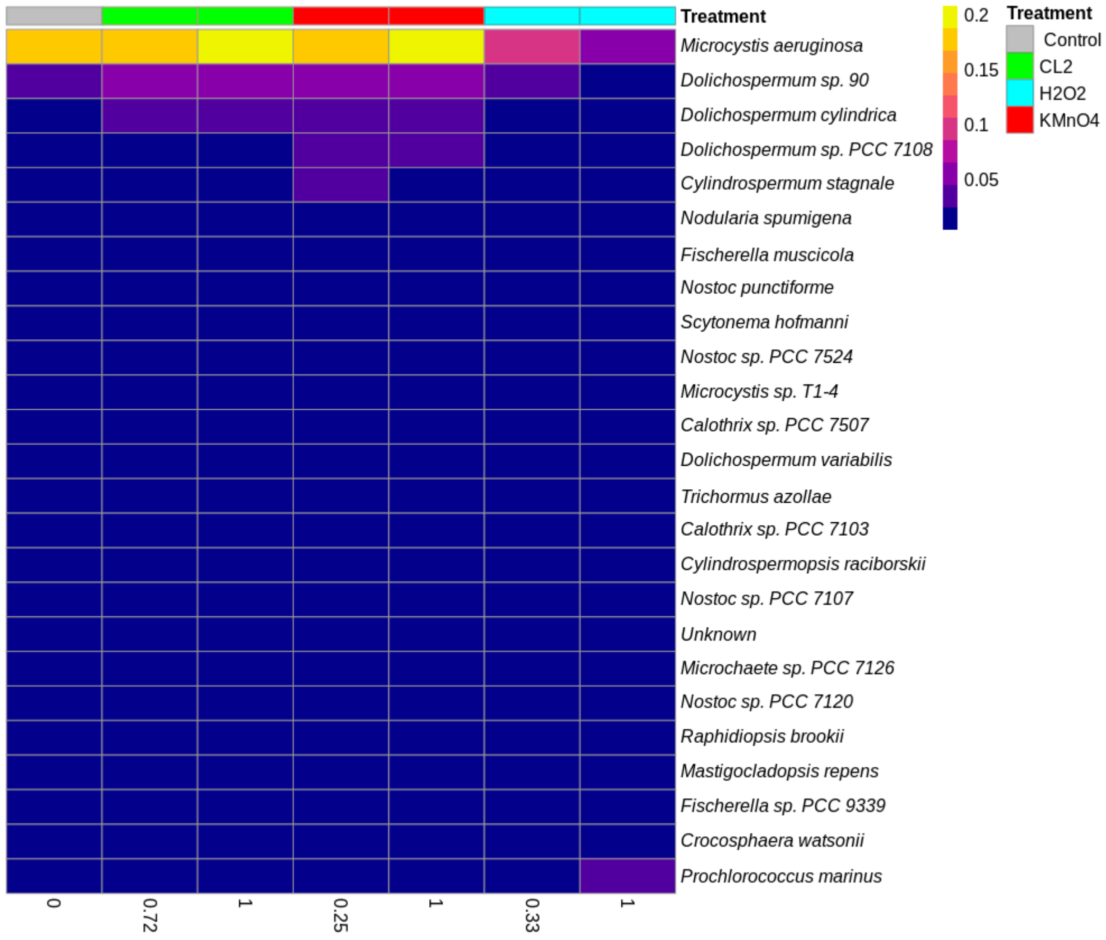

Figure S3. Cyanobacterial Species heat map following the oxidation using Cl_2, $KMnO_4$, H_2O_2 (29 August 2018 abundant: *Microcystis*).

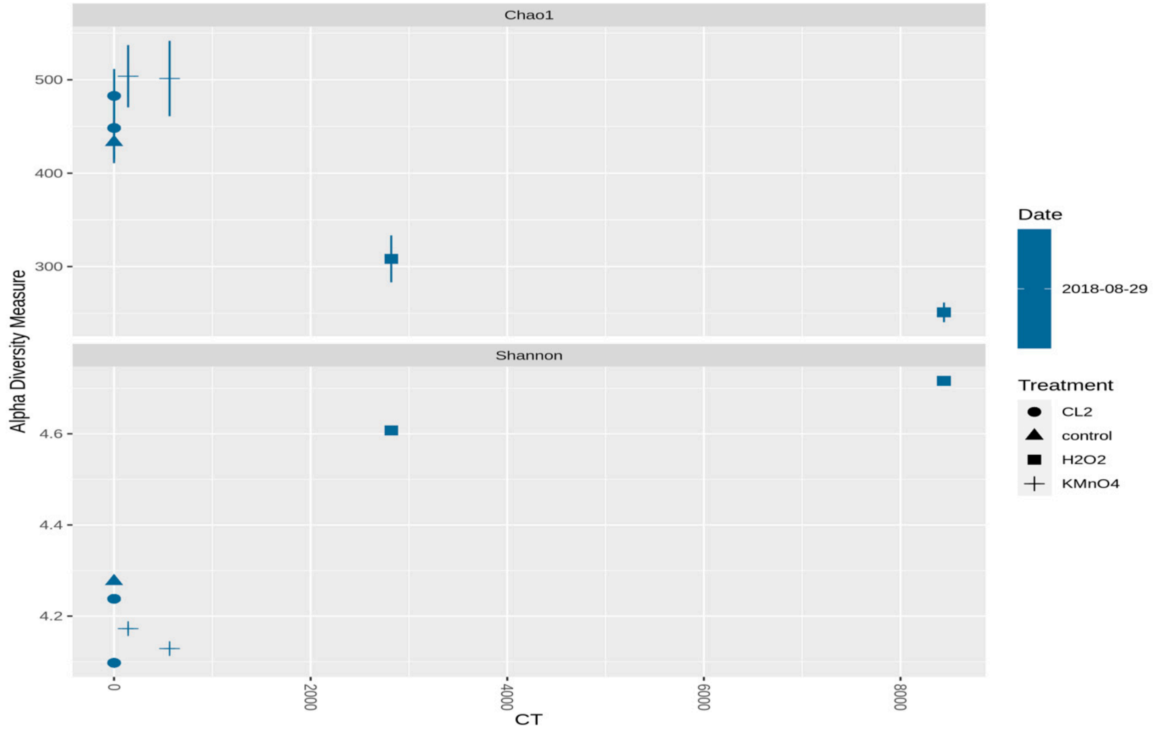

Figure S4. Alpha diversity measures of cyanobacterial community following oxidation Cl_2, $KMnO_4$, H_2O_2 (29 August 2018, abundant genus: *Microcystis*).

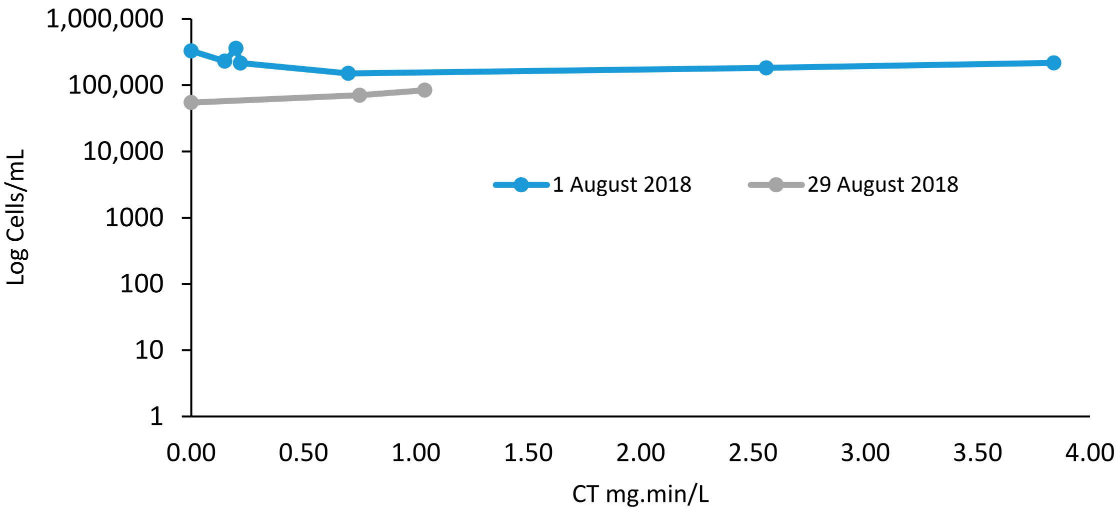

Figure S5. Total cyanobacteria cell counts following chlorination for 1 August 2018 trial and 29 August 2018 trial.

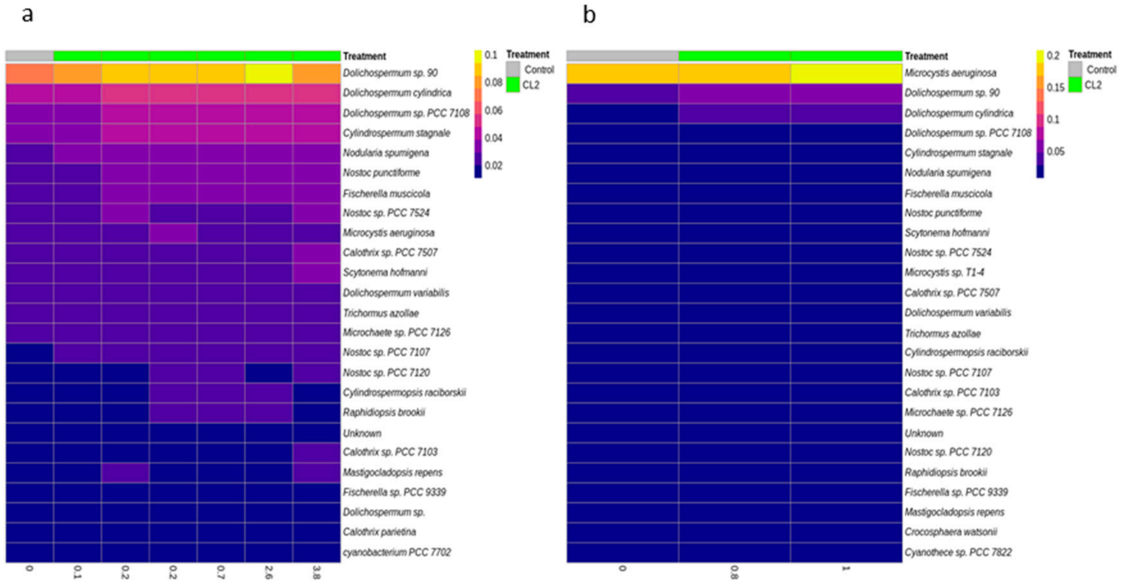

Figure S6. Cyanobacterial species heat map following the chlorination (**a**) 1 August 2018 trial, (**b**) 29 August 2018 trial.

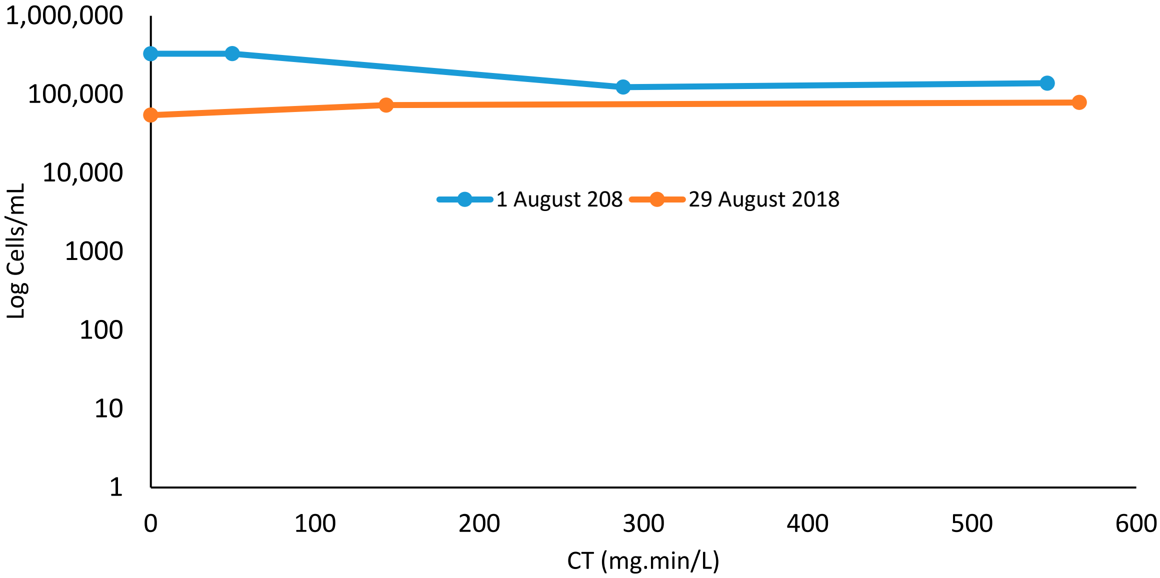

Figure S7. Total cyanobacteria cell counts following the permanganate oxidation for 1 August 2018 trial and 29 August 2018 trial.

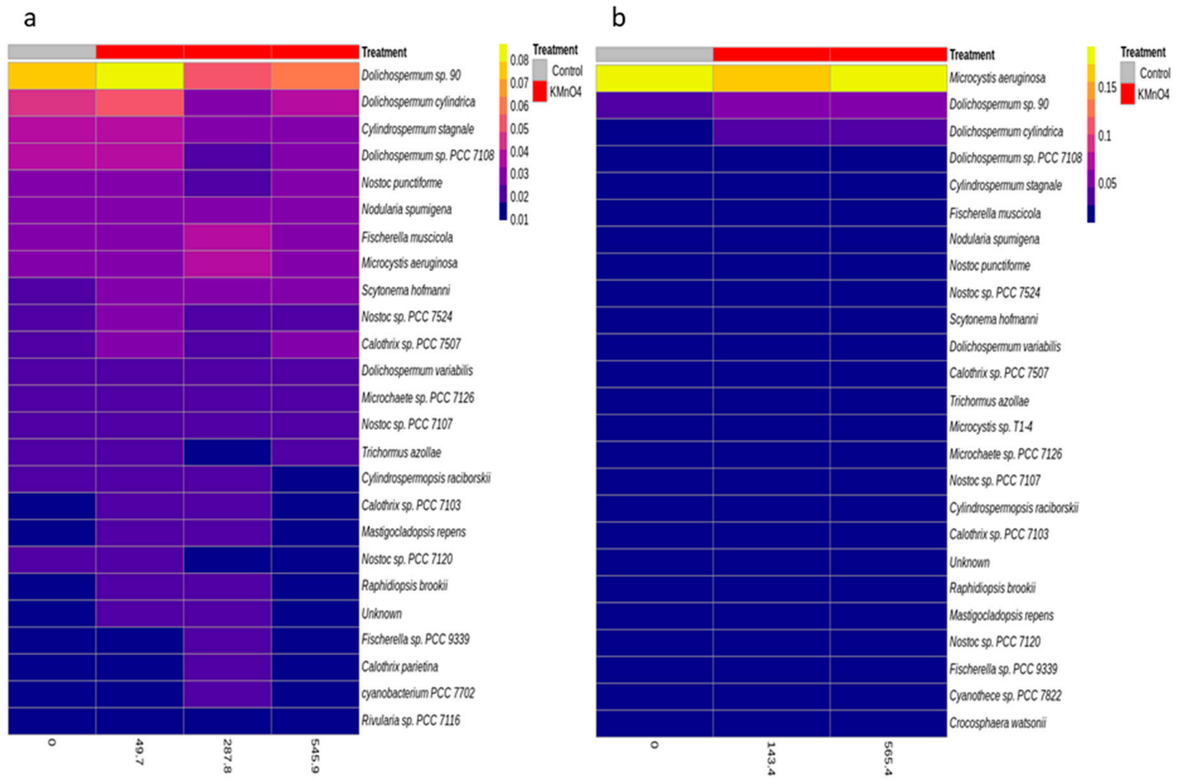

Figure S8. Cyanobacterial Species heat map following the oxidation using $KMnO_4$ (**a**) 1 August 2018 trial, (**b**) 29 August 2018 trial.

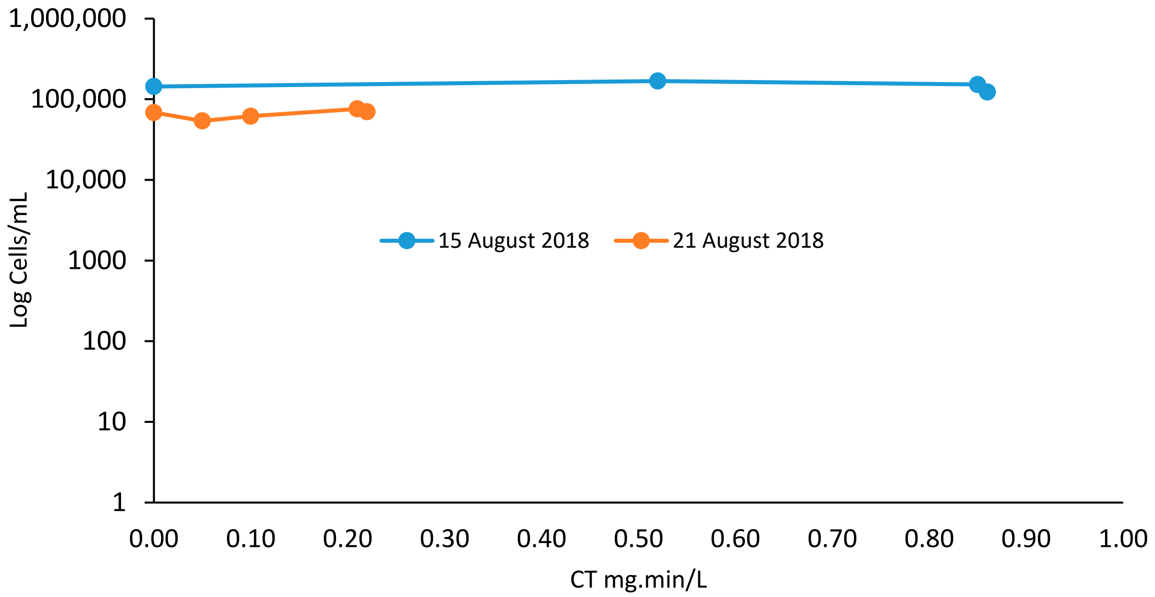

Figure S9. Total cyanobacteria cell counts following the O_3 oxidation for 15 August 2018 trial and 21 August 2018 trial.

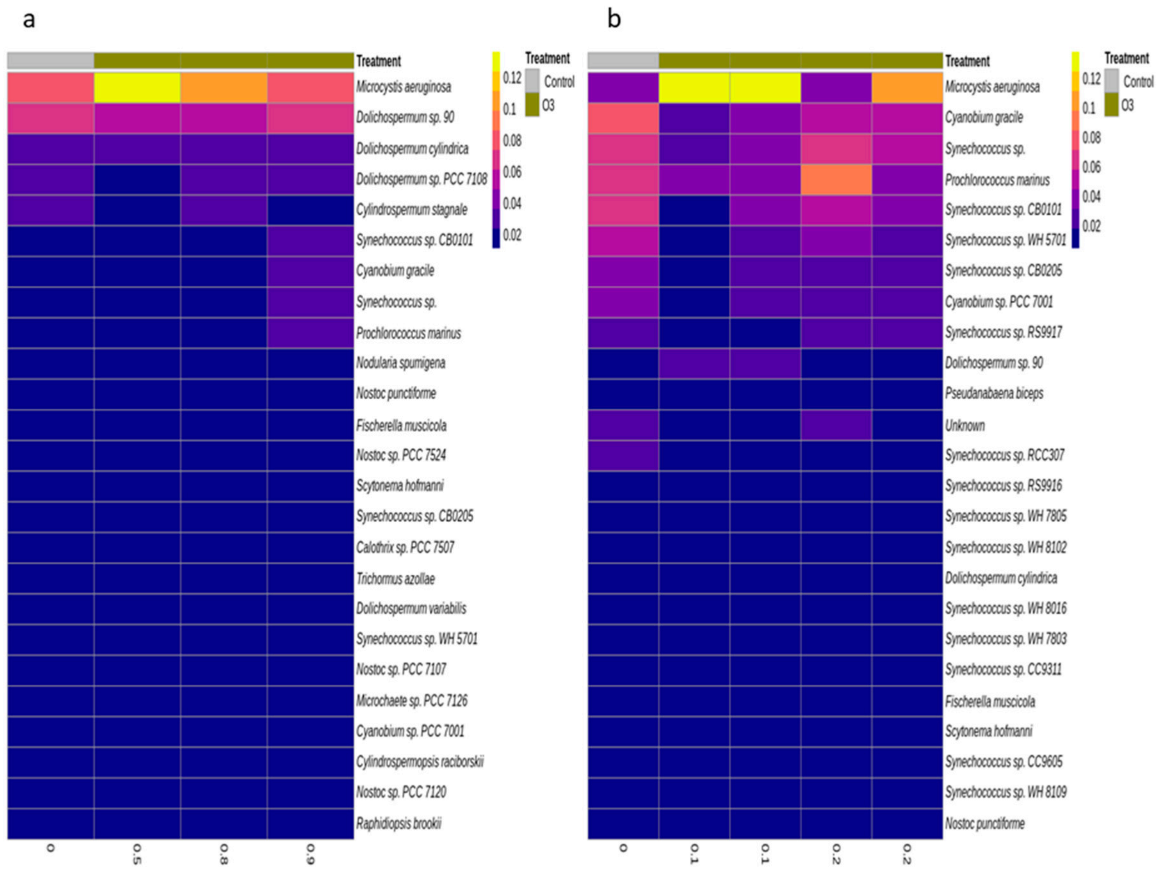

Figure S10. Cyanobacterial Species heat map following the oxidation using O_3 (**a**) 15 August 2018 trial, (**b**) 21 August 2018 trial.

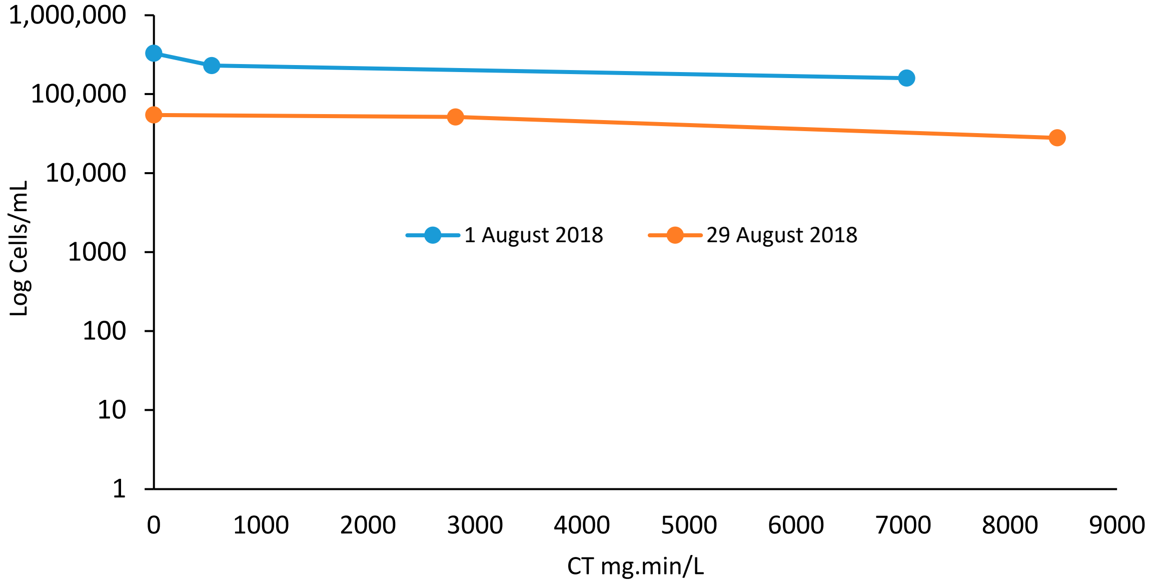

Figure S11. Total cyanobacteria cell counts following the H_2O_2 oxidation for 1 August 2018 trial and 29 August 2018 trial.

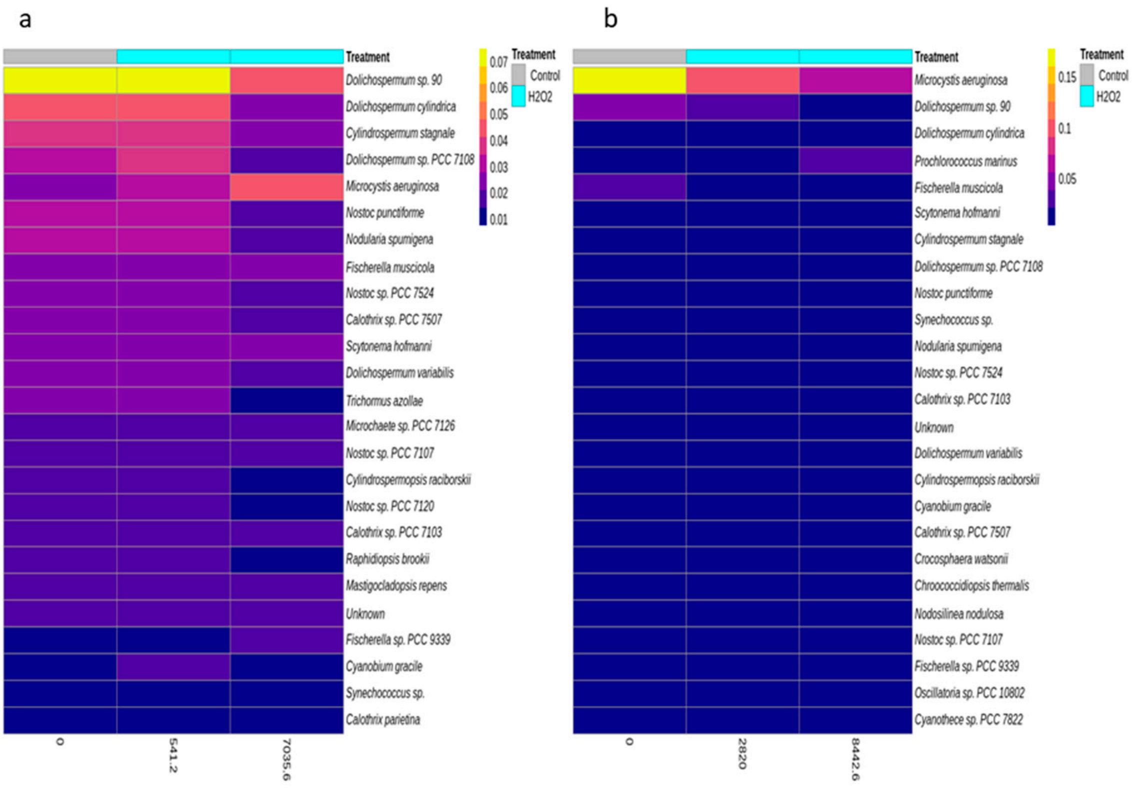

Figure S12. Cyanobacterial Species heat map following the oxidation using H_2O_2 (**a**) 1 August 2018 trial (**b**) 29 August 2018 trial.

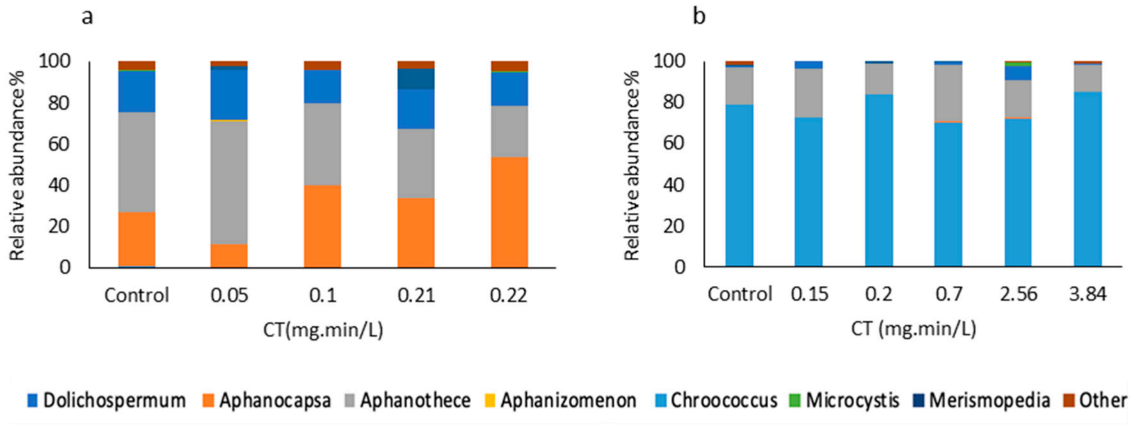

Figure S13. Relative abundance of cyanobacteria species (via light Microscopy) following oxidation (**a**) O_3 second trial (15 August 2018) (**b**) Cl_2 first trial (1 August 2018).
